# Prediction of renal cell carcinoma: Development and validation of machine learning model

**DOI:** 10.1097/MD.0000000000047205

**Published:** 2026-01-23

**Authors:** Tingjin Zheng, Rong Xu, Jianming Zhang, Yingzhi Xu, Chong Zeng, Zhishan Zhang

**Affiliations:** a Department of Clinical Laboratory, Quanzhou First Hospital Affiliated to Fujian Medical University, Quanzhou City, Fujian, China; b Department of Pharmacy, Quanzhou Medical College, Quanzhou City, Fujian, China; c Medical Research Center & Dermatology, Shunde Hospital, Southern Medical University, Foshan, Guangdong, China.

**Keywords:** clinical data, machine learning, prediction model, renal cell carcinoma

## Abstract

Renal cell carcinoma (RCC) is the leading cause of urinary system morbidity and mortality. Early identification is crucial for improving RCC patient outcomes. This study aims to construct and validate an RCC prediction model for at-risk individuals using machine learning (ML) based on routine clinical data. Data from the Quanzhou First Hospital Affiliated with Fujian Medical University between March 2014 and March 2024 were retrospectively collected, with 70% randomly assigned to the training cohort and 30% to the validation cohort. Univariate and hierarchical clustering methods were employed to identify discriminatory features to enable optimal ML algorithm selection. The performance of 7 kinds of ML algorithms-based models was evaluated based on sensitivity (recall), accuracy, F1-score, area under the receiver operating curve (AUC), discrimination, calibration, and clinical net benefit. The algorithm achieving the best AUC was selected for combination with recursive feature elimination to identify features that maximize model performance and stability. After that, the RCC prediction model was finally constructed, and the Shapley Additive Explanations method was used to visualize model characteristics and individual case predictions. Among those algorithms, the eXtreme Gradient Boosting algorithm achieving the best performance was selected for final construction. Combined with the recursive feature elimination method, it identified 21 clinically relevant variables, including age, total protein, albumin, total bilirubin, alanine aminotransferase, alkaline phosphatase, gamma-glutamyl transpeptidase, glucose, lactate dehydrogenase, creatine kinase-MB, creatinine, potassium-chloride ratio, sodium ion, calcium ion, eosinophil count, hemoglobin, platelet count, Systemic Immune-Inflammation Index, Pan-Immune-Inflammation Value, platelet–lymphocyte ratio, and sodium–chloride ratio for RCC model construction. Subsequently, a RCC prediction model and eXtreme Gradient Boosting using these 21 variables was built, achieving AUC of 0.955 (95% CI: 0.938–0.976) and an average precision of 0.923 in the validation cohort. The additional calibration curve showed high agreement between predicted and observed risks. Finally, the Shapley Additive Explanations method well demonstrated the importance of all model features and provided case-specific interpretation for clinicians. We developed and validated an ML model using routine clinical data for large-scale RCC screening. This cost-effective approach facilitates the early detection of and intervention for RCC, which may lead to improved clinical outcomes.

## 1. Introduction

Renal cell carcinoma (RCC) is one of the 3 most prevalent malignancies of the urinary system, accounting for an estimated 2% to 3% of global cancer diagnoses and mortality.^[1]^ Besides, the RCC incidence rates are increasing, particularly in developed countries. Nevertheless, RCC is typically asymptomatic in early stages, and approximately 25% to 30% of patients present with distant metastases at their initial diagnosis.^[2]^ Moreover, the prognosis for RCC varies significantly depending on tumor stage at diagnosis. While localized RCC exhibits excellent prognosis with 95% 5-year survival, metastatic disease portends a poor outcome, with dismal 10% 5-year survival and median survival under 10 months.^[3]^ Therefore, the prognosis of RCC depends largely on the stage at which the tumor is detected, early detection of RCC is critical to improve the survival of affected patients.

The classic diagnostic triad for RCC consists of flank pain, hematuria, and a palpable abdominal mass. This classic triad is present in fewer than 10% of patients at initial diagnosis and strongly correlates with advanced-stage disease, confirming its limited utility in early-stage diagnosis.^[4]^ In clinical practice, approximately 60% of RCCs are detected incidentally during abdominal imaging modalities, include ultrasonography (US), computed tomography (CT), and magnetic resonance imaging (MRI), performed for unrelated indications.^[1]^ Among them, the US is the most widely used due to its cost-effectiveness and radiation-free. However, utility as a screening tool for RCC in asymptomatic populations remains controversial, given the exceedingly low incidence of incidental malignant findings: reported as merely 0.2% in contemporary studies.^[5]^ CT and MRI provide superior diagnostic accuracy for RCC detection, but they are substantial costs and infrastructure requirements currently preclude their usage in population-wide screening programs.^[6]^

Besides that, the tissue biopsy maintains its status as the diagnostic gold standard for RCC.^[7]^ However, its invasive nature and potential risks, such as hemorrhage^[8]^ and infection, precludes its application in screening asymptomatic populations.^[9]^ Liquid biopsy focuses on blood or body secretions, making it present the individual’s health status in a minimally invasive manner.^[10]^ Moreover, its cost-effectiveness and ability for longitudinal monitoring have established an essential role in both health screening programs and clinical diagnostic testing. Therefore, multiple validated biomarkers, such as alpha-fetoprotein in hepatocellular carcinoma,^[11]^ has been established to predict health status. In RCC, several liquid biopsy biomarkers, including miR-210 and miR-196a-5p, have demonstrated promising diagnostic potential.^[12,13]^ The clinical application of liquid biopsy in RCC remains limited compared to its established utility in other tumors. Notably, to date, no liquid biopsy biomarkers have been clinically approved for RCC diagnosis.

**Figure 1.**
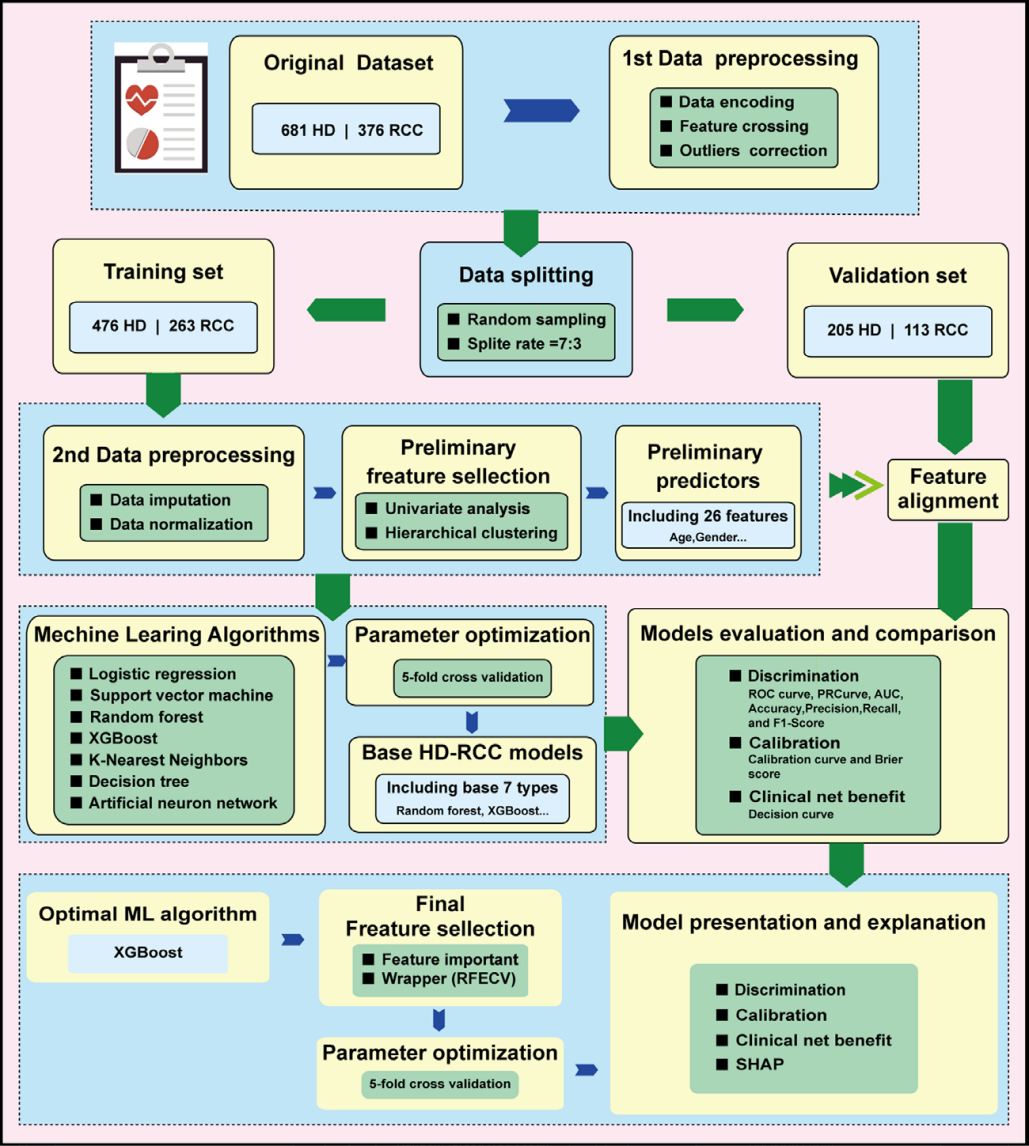
The workflow of this study.

Despite the current deficiency, many routinely measured parameters, such as serum creatinine^[14,15]^ and calcium,^[16]^ have been demonstrated closely to the RCC. However, in clinical practice, slight alterations in such parameters, especially in health check individuals, are often overlooked even when they are in the early stages of cancer. Nowadays, with the advantage of machine learning (ML) algorithms development, combining demographic data with those once-underestimated parameters may establish a reliable prediction model for RCC, as they have been determined in other disease.^[17]^ Therefore, the aim of this study is to develop and validate a cost-effective, large-scale screening ML-based model using routine clinical data for early detection of RCC. The workflow of this study is shown in Figure 1.

## 2. Materials and methods

### 2.1. Participants

Clinical data of 376 pathologically diagnosed RCC patients and 681 healthy controls (HC) individuals were admitted to the Quanzhou First Hospital Affiliated to Fujian Medical University from March 2014 to March 2024 were retrospectively collected. The study was approved by the Ethics Committee of the Quanzhou First Hospital Affiliated to Fujian Medical University, and informed consent was waived because of the retrospective nature of the study (No. 2025-K224). The inclusion and exclusion criteria were as follows: RCC patients: first, age exceeding 18 years, regardless of gender; second, pathological findings confirmed the diagnosis of RCC; third, absence of prior clinical treatment for RCC disease prior to admission. No RCC supportive therapy, such as medication or other therapeutic interventions, was permitted until study enrollment; fourth, RCC diagnosis must align with the diagnostic criteria stipulated in the “Standard for Diagnosis and Treatment of RCC (2019 Edition)”^[18]^; fifth, availability of blood routine and biochemical test data; sixth, the patients who missing clinical data were excluded. HC individuals: first, no family history of cancer and RCC-related disease; second, routine tests including blood routine and biochemical; third, missing or incomplete clinical data were excluded.

### 2.2. Data pre-processing

The dataset for this study initially consisted of 55 features, including 46 continuous variables and 9 categorical ones. Data outliers in continuous variables were identified using box plots. An outlier was defined as any value exceeding the upper quartile (Q3) by more than 1.5 times the interquartile range (IQR) or falling below the lower quartile (Q1) by more than 1.5 times the IQR. Identified outliers were winsorized; each was replaced by the nearest boundary value (Q1 − 1.5IQR or Q3 + 1.5IQR) to reduce their impact while preserving the overall distribution of the main data.

To avoid data leakage, participants were randomly allocated to training (70%) and validation (30%) cohorts using Scikit-learn package. Features with missing rates exceeding 30% were excluded to prevent prediction bias. Missing values in retained features (<30% missingness) were imputed using cohort-specific feature means (training cohort means for training set imputation; validation cohort means for validation set imputation). Features in the training cohort were analyzed using univariate methods, and those showing statistically significant differences between HC and RCC (*P* < .05) were retained.

Hierarchical clustering with average linkage (distance threshold = 0.3; equivalent to |*R*| > 0.7) was then applied to the feature correlation matrix to eliminate redundant features and reduce multicollinearity.

### 2.3. Modeling algorithms selection

Different algorithms possess distinct feature optimization strengths, making algorithm selection critical for prognostic model construction. Therefore, in this study 7 ML algorithms, including Logistic Regression (LR), Decision Tree (DT), Random Forest (RF), Extreme Gradient Boosting (XGBoost), K-Nearest Neighbor classification (kNN), Support Vector Machine (SVM), and Artificial Neural Network (ANN), were implemented to build base models and subsequently compared for optimal algorithm selection. Five-fold cross-validation was used to ensure the stability of the ML-based model. We used grid search to select the best hyperparameters for each algorithm during tuning. In parameter tuning process, the model achieving the highest area under the curve (AUC) of receiver operating characteristic, namely as AUC, was selected as optimal. Subsequently, the models were validated and compared on the validation set. We assessed discrimination metrics, including sensitivity (recall), accuracy, F1-score, and AUC. Given that AUC served as the primary selection criterion, the best-performing algorithm was chosen for final model construction. Moreover, we evaluated the discrimination, calibration, and clinical net benefit of the models developed with these 7 ML algorithms.

### 2.4. Feature sub-selection

Recursive feature elimination (RFE) is a mainstream ML feature selection method that removes unimportant features to identify optimal feature combinations, achieving peak model performance. In this study, RFE was employed to identify optimal features maximizing XGBoost’s prediction performance and stability. Furthermore, 10 rounds of 5-fold cross-validation ensured rigorous feature selection within the XGBoost–RFE pipeline.

### 2.5. Model interpretation

ML makes it difficult to explain the contribution of each feature due to its black-box nature, so the Shapley Additive Explanations (SHAP) algorithm was introduced in this study. This is a unified framework for explaining outputs of any ML mode. Using a tree explainer adapted for SHAP, feature attributions were assessed by conceptualizing each input feature as a player in a cooperative game where model predictions constitute payouts. Within this paradigm, SHAP values quantify each feature’s importance to predictions. Feature ranking was established through SHAP importance scores, calculated as the mean absolute value of Shapley values across the dataset. Summary plots visualize both feature importance and directional effects: each point represents the SHAP value for a feature-instance pair, while positional clustering along the x-axis indicates impact on diagnostic outcomes. Complementary enforce plots further elucidate feature attributions for individual predictions.

### 2.6. Statistical analysis

In statistical description terms, continuous variables were described as the mean (SD) or median (IQR) as appropriate. Correlations were determined by Pearson analysis. Univariate analysis was performed using the *t* test or chi-square test. Statistical significance was defined as a 2-sided *P* value < .05. All data were analyzed using Python (version 3.10.8; https://www.python.org).

## 3. Results

### 3.1. Baseline characteristics

A total of 1057 participants were admitted into this study from January 2014 to December 2024. Among the participants, 681 were health check individuals, while 376 were diagnosed with RCC. In this study, 57 characteristics, including 2 demographic characteristics and 55 clinical characteristics, were collected (Table S1, Supplemental Digital Content, https://links.lww.com/MD/R159). The matrix plot showcased that 8 of them have missing rates exceeding 30%, which were removed in the next analysis (Figure S1, Supplemental Digital Content, https://links.lww.com/MD/R160).

After that, the demographic and clinical data of the participants are presented (Table S2, Supplemental Digital Content, https://links.lww.com/MD/R159). Compared to the HC group, the RCC group had significantly higher proportions of patients with elevated levels in 24 features, including alkaline phosphatase (ALP), gamma-glutamyl transpeptidase (GGT), glucose (GLU), creatinine (CREA), and white blood cell count, and with reduced levels in 19 features, such as total protein, albumin (ALB), and alanine aminotransferase (*P* < .05; Table 1).

**Table 1:** Comparison of HC and RCC for all features.

Features	HC (n = 681)	RCC (n = 376)	*t*/*χ*^2^ value	*P* value
Gender, n (%)			8.2107	.004
Female	289 (42.44)	125 (33.24)		
Male	392 (57.56)	251 (66.76)		
Age (yr)	43.34 ± 12.17	57.62 ± 12.57	-17.89	<.001
TP (g/L)	74.99 ± 3.80	71.06 ± 7.45	9.57	<.001
ALB (g/L)	44.27 ± 2.60	40.67 ± 5.33	12.32	<.001
GLO (g/L)	30.73 ± 3.13	30.34 ± 5.76	1.22	.225
TBIL (µM)	13.65 ± 5.28	14.30 ± 7.18	-1.54	.123
DBIL (µM)	2.50 ± 1.02	2.97 ± 2.96	-3.00	.003
IBIL (µM)	11.15 ± 4.38	11.50 ± 5.83	-0.99	.320
AST (U/L)	25.79 ± 11.47	25.63 ± 35.01	0.09	.930
ALT (U/L)	28.56 ± 20.16	24.79 ± 27.60	2.32	.020
ALP (U/L)	73.24 ± 21.61	91.25 ± 95.62	-3.60	<.001
GGT (U/L)	31.66 ± 33.88	42.43 ± 58.70	-3.27	.001
GLU (mM)	5.58 ± 1.66	6.05 ± 2.24	-3.54	<.001
LDH (U/L)	169.90 ± 32.37	186.12 ± 118.78	-2.59	.010
CK (U/L)	112.45 ± 110.97	104.41 ± 108.37	1.15	.252
CKMB (U/L)	11.36 ± 5.98	13.30 ± 8.53	-3.92	<.001
CREA (µM)	66.73 ± 16.47	88.94 ± 98.36	-4.34	<.001
UA (µM)	362.60 ± 95.56	372.02 ± 107.10	-1.42	.156
K (µM)	4.30 ± 0.33	4.11 ± 0.44	7.50	<.001
Na (µM)	140.07 ± 1.77	139.10 ± 2.85	6.01	<.001
Cl (µM)	103.34 ± 2.08	103.73 ± 3.26	-2.13	.034
Ca (µM)	2.39 ± 0.09	2.33 ± 0.14	7.17	<.001
Mg (µM)	0.90 ± 0.06	0.87 ± 0.08	4.97	<.001
P (µM)	1.23 ± 0.55	1.18 ± 0.24	1.96	.050
WBC (10^9^/L)	6.67 ± 1.79	7.22 ± 2.68	-3.56	<.001
NEUT (10^9^/L)	3.78 ± 1.39	4.68 ± 2.46	-6.55	<.001
LYMP (10^9^/L)	5.80 ± 8.98	1.76 ± 0.63	11.66	<.001
MONO (10^9^/L)	5.72 ± 13.79	0.71 ± 3.28	9.04	<.001
EO (10^9^/L)	0.18 ± 0.23	0.13 ± 0.14	4.47	<.001
BASO (10^9^/L)	0.04 ± 0.04	0.04 ± 0.21	0.02	.981
RBC (10^12^/L)	15.50 ± 28.24	4.53 ± 0.76	10.12	<.001
HGB (g/L)	159.91 ± 48.34	133.80 ± 22.21	11.99	<.001
HCT (L/L)	56.08 ± 34.77	39.59 ± 6.37	12.02	<.001
MCV (fL)	83.66 ± 16.18	87.95 ± 6.19	-6.15	<.001
MCH (pg)	66.68 ± 97.72	30.46 ± 16.28	9.44	<.001
MCHC (g/L)	293.46 ± 108.26	334.07 ± 30.06	-9.17	<.001
RDW (%)	35.42 ± 12.82	41.57 ± 5.37	-10.92	<.001
PLT (10^9^/L)	230.09 ± 103.63	252.74 ± 83.53	-3.87	<.001
SIRI	1.51 ± 2.04	2.10 ± 4.36	-2.48	.014
SII	382.01 ± 266.45	815.42 ± 855.83	-9.57	<.001
PIV	202.56 ± 175.17	534.06 ± 1052.93	-6.06	<.001
PLR	101.22 ± 53.48	161.35 ± 82.14	-12.78	<.001
NLR	1.46 ± 0.88	3.26 ± 3.53	-9.71	<.001
CaP	2.95 ± 1.37	2.76 ± 0.57	3.09	.002
CaPR	2.00 ± 0.27	2.04 ± 0.40	-1.57	.116
CaMgR	2.67 ± 0.22	2.69 ± 0.29	-0.91	.366
NaKR	32.74 ± 2.46	34.24 ± 3.64	-7.16	<.001
NaClR	1.36 ± 0.03	1.34 ± 0.03	7.48	<.001
KClR	0.04 ± 0.00	0.04 ± 0.00	7.57	<.001

ALB = albumin, ALP = alkaline phosphatase, ALT = alanine aminotransferase, AST = aspartate aminotransferase, BASO = basophil count, Ca = calcium ion, CaMgR = calcium–magnesium ratio, CaP = calcium–phosphorus product, CaPR = calcium–phosphorus ratio, CK = creatine kinase, CKMB = creatine kinase-MB, Cl = chloride ion, CREA = creatinine, DBIL = direct bilirubin, EO = eosinophil count, GGT = gamma-glutamyl transpeptidase, GLO = globulin, GLU = glucose, HCT = hematocrit, HGB = hemoglobin, IBIL = indirect bilirubin, K = potassium ion, KClR = potassium–chloride ratio, LDH = lactate dehydrogenase, LYMP = lymphocyte count, MCH = mean corpuscular hemoglobin, MCHC = mean corpuscular hemoglobin, MCV = mean corpuscular volume, Mg = magnesium ion, MONO = monocytes count, Na = sodium ion, NaClR = sodium-chloride ratio, NaKR = sodium–potassium ratio, NEUT = neutrophil count, NLR = neutrophil–lymphocyte ratio, P = phosphorus ion, PIV = Pan-Immune-Inflammation Value, PLR = platelet–lymphocyte ratio, PLT = platelet count, RBC = red blood cell count, RDW = red blood cell distribution width, SII = Systemic Immune-Inflammation Index, SIRI = System Inflammation Response Index, TBIL = total bilirubin, TP = total protein, UA = uric acid, WBC = white blood cell count.

To avoid data leakage, the participants were randomly divided into training and validation cohorts at a 7:3 ratio. Subsequently, the features with >70% completeness had missing values were imputed by mean replacement (Table S3, Supplemental Digital Content, https://links.lww.com/MD/R159).

### 3.2. Optimal ML algorithm selection

**Figure 2.**
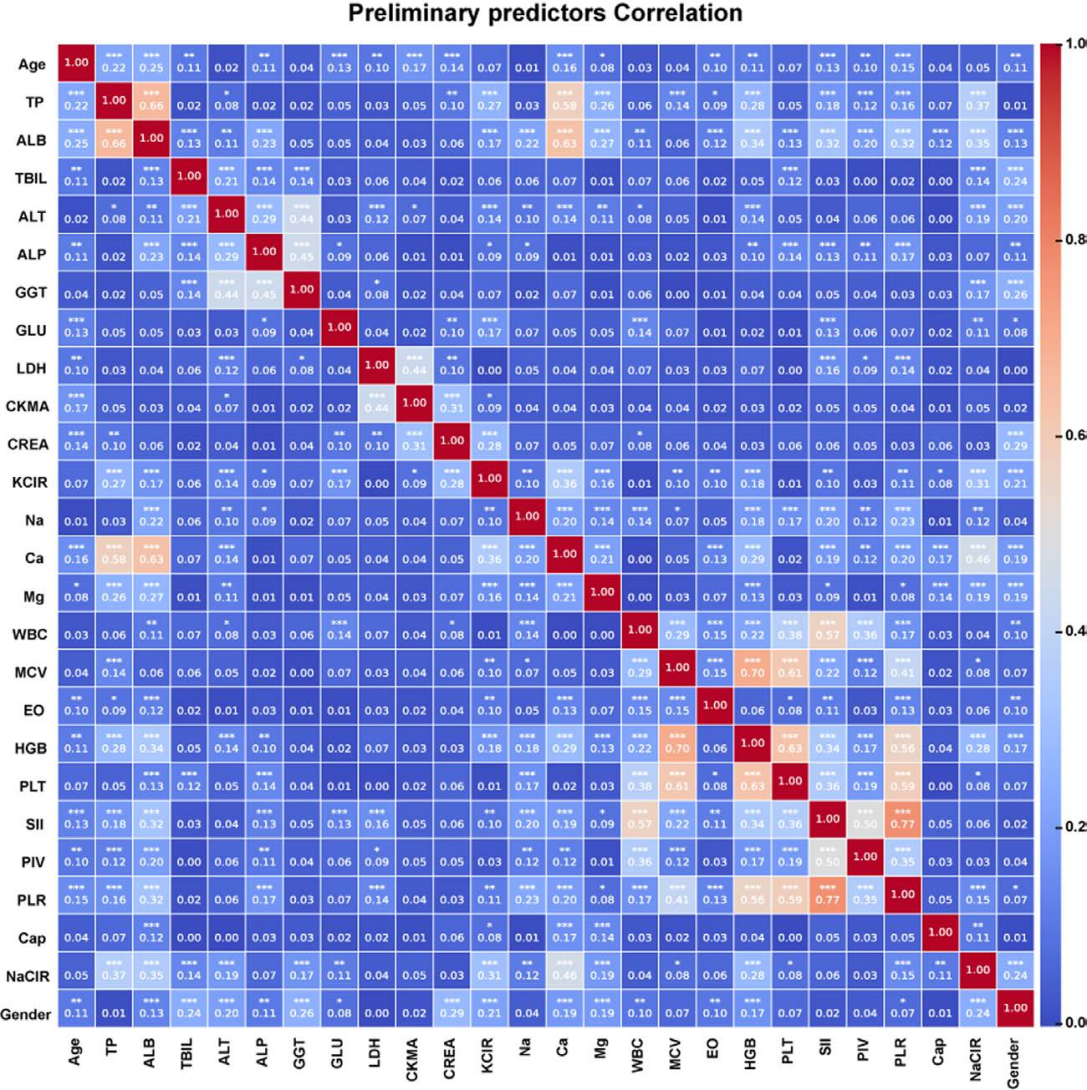
Correlation among remaining clinical features after removing highly correlated ones.

**Figure 3.**
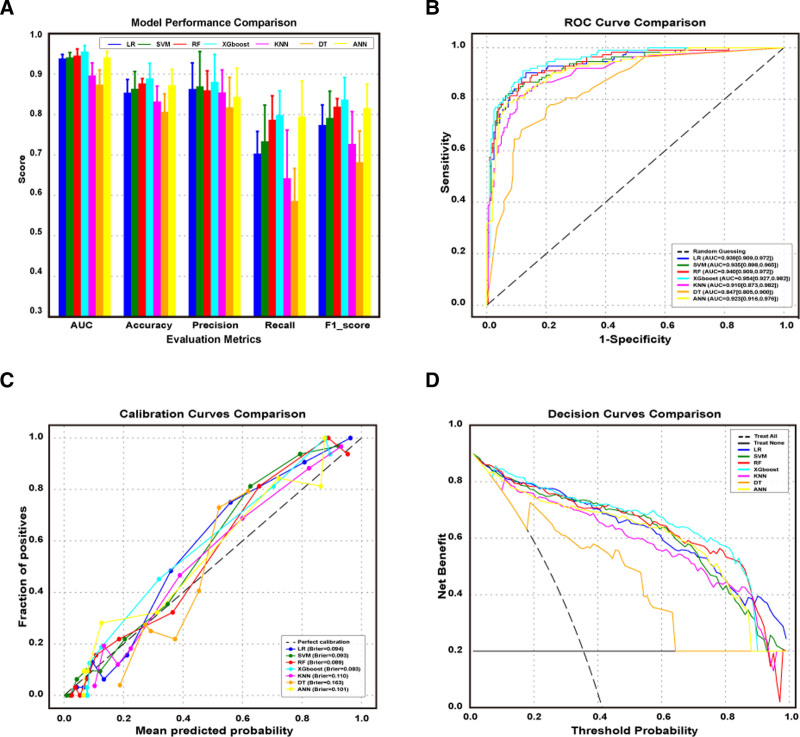
Comprehensive performance evaluation of 7 ML algorithms. (A) Metrics box plot for ML algorithms. (B) Receiver operating characteristic curves for ML algorithms. (C) Calibration curves of ML algorithms. (D) Decision curves of ML algorithms. ML = machine learning.

The univariate analysis, including independent-samples *t* tests and *χ*² tests, were performed to extracted the statistically significant features within the training cohort between the HC and RCC groups (Table S4, Supplemental Digital Content, https://links.lww.com/MD/R159). The corresponding correlations between all variables within training cohort were then calculated. The correlation heatmap revealed that numerous features exhibited strong pairwise Pearson correlations (e.g., red blood cell count and lymphocyte count, |*r*| = 0.99), suggesting potential collinearity among these features (Figure S2A, Supplemental Digital Content, https://links.lww.com/MD/R160). Therefore, the hierarchical clustering algorithm was employed to auto identify feature clusters and eliminate redundant features within each cluster based on training cohort (Figure S2B, Supplemental Digital Content, https://links.lww.com/MD/R160). As illustrated in Figure 2, 26 features detailed in Table S5, Supplemental Digital Content, https://links.lww.com/MD/R159 were preliminarily selected after removing collinear features to optimize ML algorithm selection. To achieve this, the preliminary models based on 7 ML algorithms, including LR, SVM, RF, eXtreme Gradient Boosting (XGBoost), KNN, DT, and ANN, were constructed. We also present performance metrics, including AUC, accuracy, precision, recall, and F1 score, for the preliminary HC–RCC models developed based on validation cohort. XGBoost significantly outperformed other models, achieving 89.3% accuracy (Fig. 3A), which underscores its robustness in classification tasks. It also achieved the highest AUC value of 0.954 (95% CI: 0.927–0.982), making it the most effective model in distinguishing between the groups (Fig. 3B). Closely following, the RF model also demonstrated strong performance, with an accuracy of 89.3% and an AUC of 0.940 (95% CI: 0.909–0.972). In contrast, the DT model shows the weakest performance, with the lowest accuracy of 79.2 % and a F1 score of 0.663. Furthermore, there are significant statistical differences in the metrics of the different models.

Besides, the calibration and clinical net benefit of those algorithms on the validation cohort were calculated. The calibration curve describes that the XGBoost algorithm achieved the excellent agreement between predicted and observed outcomes among all algorithms, with the lowest Brier score (0.083) (Fig. 3C). This suggests no deviation from an ideal fit. Further, decision curves demonstrated that the XGBoost algorithms offers a superior net benefit for predicting RCC compared to the “treat all or none” strategy across most risk thresholds (Fig. 3D). In summary, these results suggest that the XGBoost algorithms is optima for current clinical RCC models constructio.

### 3.3. XGBoost based RCC model construction

**Figure 4.**
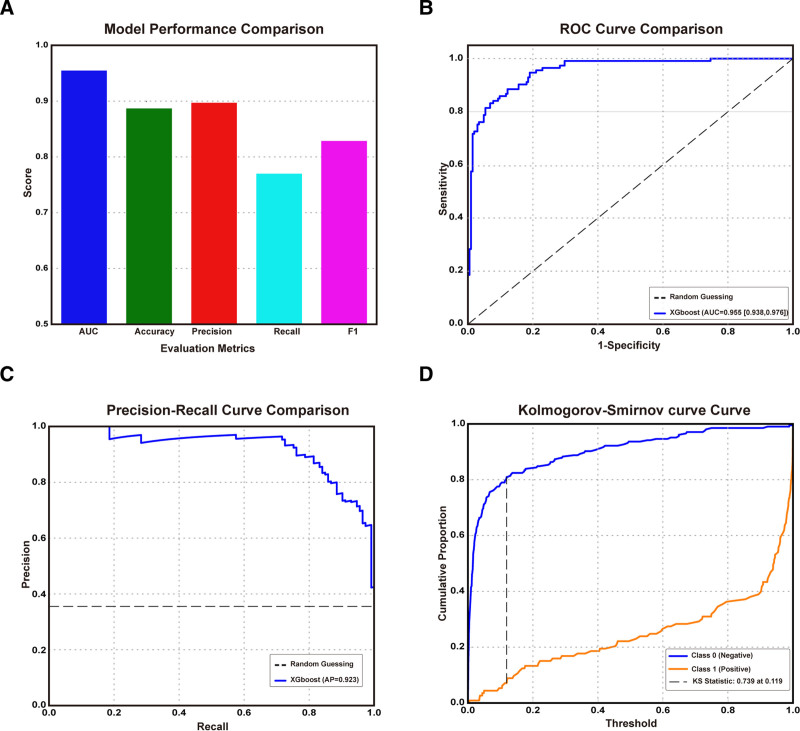
Comprehensive performance evaluation of XGBoost model in predicting HC and RCC. (A) Metrics of the models. (B) ROC curves for models. (C) Precision-recall curves of models. (D) Kolmogorov–Smirnov curves of models. HC = healthy controls, RCC = renal cell carcinoma, ROC = receiver operating characteristic curve, XGBoost = eXtreme Gradient Boosting.

RFE is a well-established feature selection technique that enhances model performance and computational efficiency by retaining only the most informative predictors. To optimize our RCC risk prediction model, we implemented a RFE algorithm coupled with XGBoost (XGBoost–RFE) for robust feature selection. Applied to the training cohort, this approach identified 21 clinically relevant variables, including age, total protein, ALB, total bilirubin, alanine aminotransferase, ALP, GGT, GLU, lactate dehydrogenase, creatine kinase-MB, CREA, potassium-chloride ratio, sodium ion, calcium ion, eosinophil count, hemoglobin, platelet count, Systemic Immune-Inflammation Index, Pan-Immune-Inflammation Value, platelet–lymphocyte ratio, and sodium–chloride ratio, as predictors for RCC model construction (Table S6, Supplemental Digital Content, https://links.lww.com/MD/R159; Figure S3, Supplemental Digital Content, https://links.lww.com/MD/R160). Subsequently, a comprehensive grid search was conducted to determine the optimal hyperparameters for XGBoost architecture. As shown in Figure 4A and B, the performance of the XGBoost model on the validation cohort was initially assessed, with achieving accuracy at 88.7%, the AUC of 0.955 (95% CI: 0.938–0.976) and the F1 score at 0.826, indicating the excellent of model. Further precision-recall curves, with average precision = 0.923, also highlighting its superior ability in varying precision-recall conditions (Fig. 4C). Additional Kolmogorov–Smirnov curves, with KS value 0.694 at 0.199, reclaim the satisfactory of current model (Fig. 4D), as well. These results validate the model’s effectiveness and highlight its potential clinical utility in screening and identifying the individuals at high RCC risk. Collectively, the model’s validated performance metrics and biological interpretability support its potential as a clinically actionable tool for high-risk RCC population screening.

### 3.4. Optimal model interpretability

**Figure 5.**
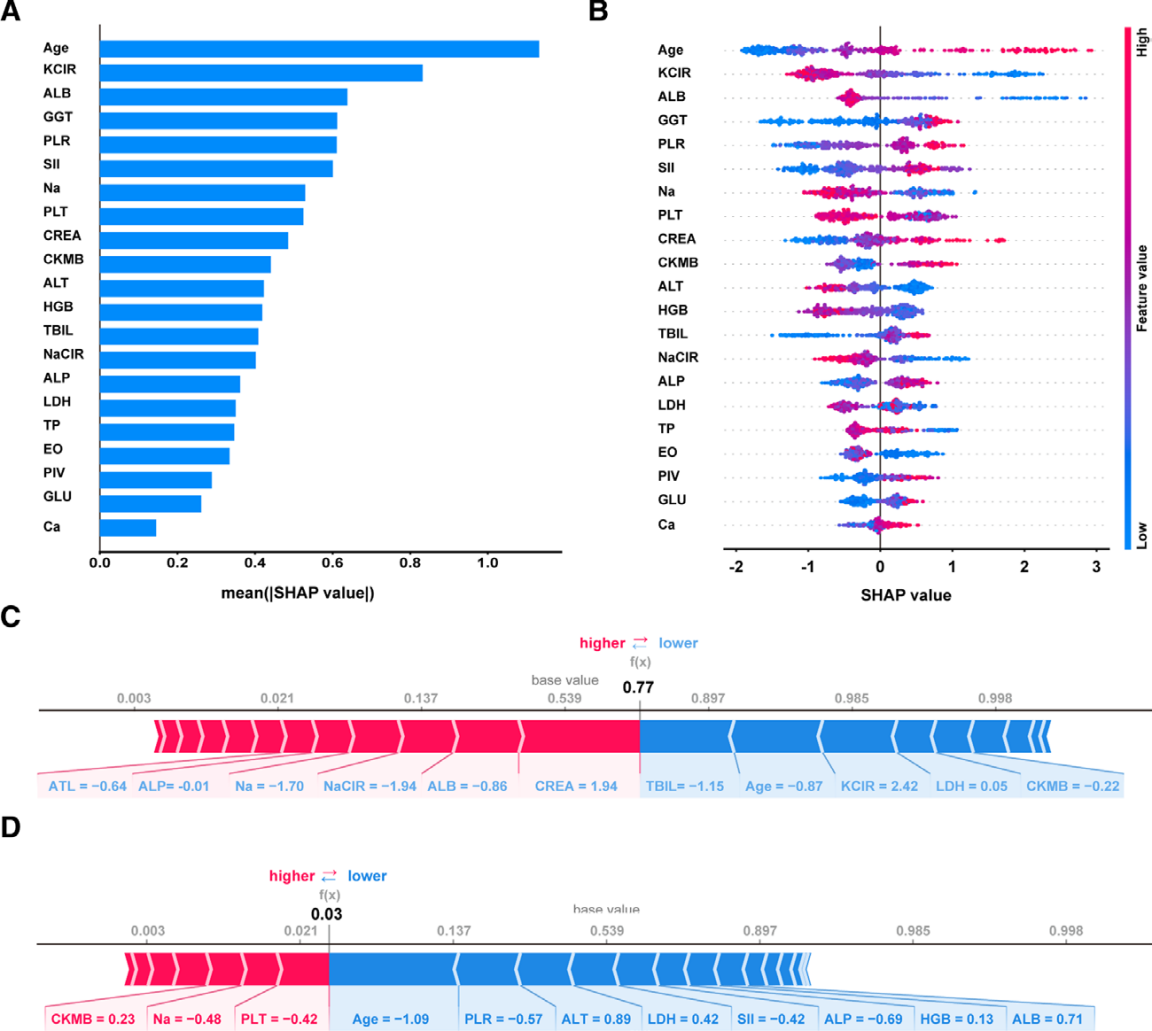
Complex machine learning model SHAP value analysis. (A) The importance ranking of features based on the mean (|SHAP value|). (B) A summary plot of the SHAP values for each feature. (C) SHAP force plot of 1 enrolled RCC patient, the model generated a predicted RCC probability of 77%. (D) SHAP force plot for 1 enrolled healthy individual, the model generated a predicted RCC probability of 3%. RCC = renal cell carcinoma, SHAP = Shapley Additive Explanations.

Given that clinicians are often hesitant to accept prediction models that lack direct explainability and interpretability, the SHAP method was utilized to interpret the output of the XGBoost model by quantifying the contribution of the 21 variables to the prediction. As showcased in Figure 5A, the age, potassium–chloride ratio, ALB, GGT, and platelet–lymphocyte ratio emerged as the top 5 most influential variables, significantly affecting the model’s predictive power. Figure 5B clearly illustrates the strength and direction of feature contributions to RCC prediction. Elevated levels of Age, GGT, platelet–lymphocyte ratio, Systemic Immune-Inflammation Index, CREA, creatine kinase-MB, total bilirubin, ALP, Pan-Immune-Inflammation Value, GLU, and calcium ion were associated with an increased risk of RCC, while other factors appeared to have a protective effect. For local interpretability, the SHAP force plot provides sample-specific explanation, demonstrating how our current model makes clinical decisions for individual cases. Each feature’s SHAP value acts as a force that either increases (red) or decreases (blue) the overall prediction score, ultimately determining the risk of RCC. For instance, in Figure 5C, 1 individual received a positive prediction of RCC with a probability of 77%, whereas in Figure 5D, another individual had a negative prediction with a probability of 3%.

## 4. Discussion

RCC remains the most frequently diagnosed adult malignancy in the urinary system,^[19]^ with an approximate 2.0% annual increase worldwide.^[20]^ Early identification of RCC is essential for effective treatment and improved outcomes, but there are several reasons that make this process complex and challenging. Notably, current diagnostic methods, including US, CT, and MRI, have limitations in sensitivity, specificity, or accessibility. Over 60% of RCC patients diagnosed incidentally,^[21]^ with one-fifth of them already presenting at higher tumor grades.^[4]^ This clinical reality underscores the urgent need for accessible RCC diagnosis prediction models.

Recently, various ML algorithms have been developed, enabling machines to solve complex problems across diverse domains without explicit programming, efficiently.^[22,23]^ In medicine, these techniques have demonstrated transformative potential across multiple areas, including clinical management,^[24]^ drug design^[25]^ and delivery,^[26]^ disease diagnosis,^[27]^ and classification, as well as prognostic prediction. With then on, numerous ML-based models have been developed for the diagnosis of RCC diagnosis. For instance, Fenstermaker et al developed a histopathology-based diagnostic model using whole-slide images from The Cancer Genome Atlas,^[28]^ while Cai et al achieved comparable performance with a complementary whole-slide images-based framework from a hospital in southern China.^[29]^ Beyond imaging, Bifarin et al pioneered a urine metabolomics approach enhanced by ensemble ML,^[30]^ and Xu et al recently introduced an integrated clinical-proteomic model for simultaneous diagnostic and prognostic assessment.^[31]^ However, despite this growing number of RCC diagnostic models, none have yet proven suitable for population-level screening. A critical limitation lies in their dependence on either invasive pathological biopsies or specialized analytical platforms that are inaccessible in routine clinical practice.

To address the current limitations, the 57 common candidate variables covering demographic information and routine blood test results were taken into consideration, initially. And the 26 preliminary predictors, identified by univariate analysis and hierarchical clustering, were applied to determine the algorithm for model building. In this study, 7 types of ML algorithms, including LR, SVM, RF, XGBoost, KNN, DT, and ANN, were systematically evaluated and comparison. Our current research demonstrates that the XGBoost algorithm exhibited superior discriminative performance compared to the others, indicating, indicating its optimality. Indeed, the XGBoost algorithm has achieving high efficiency and stability in constructing disease prediction models.^[32]^ Beyond the algorithm itself, robust features are also essential for ensuring the stability of predictive models.^[33]^ Therefore, the RFE algorithm, an excellent feature selection algorithm, was employed to reduce feature dimensionality while preserving model robustness. Finally, by incorporating the RFE algorithm, we built an XGBoost model comprising 0.955 on the independent test set.

Despite their accuracy, the black-box nature of ML models limits their interpretability,^[34]^ posing challenges for clinicians who require explainable decision-making in medical practice. To address this challenge, various methods have recently been designed to interpret ML models. In this study, SHAP, a unified model interpretation framework that has significant effectiveness in explaining XGBoost models,^[35,36]^ was employed to demonstrate how individual characteristics influenced the model’s risk predictions. With the advantage of SHAP, the contribution of each variable in our RCC diagnosis model was visualized. Importantly, the SHAP force plots provided case-specific explanations to support diagnostic decisions. Overall, this study effectively demonstrated the capability of XGBoost model to accurately predict RCC using routine clinical testing parameters.

However, there are some limitations to consider in this work. First, as a retrospective study, it may be subject to inherent selection bias despite the implementation of stringent inclusion and exclusion criteria. Second, the model was constructed based on single-center data from Quanzhou First Hospital Affiliated to Fujian Medical University. Further research is needed to include larger sample sizes from multiple centers, covering diverse geographic regions and racial backgrounds. Third, considering the generalizability of the model, the variables included in this study were all routine items that are easy to obtain in clinical practice. Some novel molecular markers associated with RCC pathogenesis, such as ciRS-7^[37]^ and miR-196a-5p,^[38]^were excluding in the present study. Nevertheless, these limitations do not diminish the robust predictive performance of our final model.

In conclusion, we successfully developed and validated a large-scale screening model to predict the RCC based on the clinical data extracted from the Quanzhou First Hospital Affiliated to Fujian Medical University. This reliable and accessible ML model demonstrates strong potential for population-wide RCC screening. With these advantages, this model may facilitate earlier therapeutic intervention during more treatable stages, thereby achieving inspiring outcomes while relieving the healthcare and economic burden.

## Author contributions

**Conceptualization:** Jianming Zhang, Yingzhi Xu, Chong Zeng, Zhishan Zhang.

**Data curation:** Tingjin Zheng, Rong Xu, Jianming Zhang, Yingzhi Xu, Chong Zeng.

**Formal analysis:** Jianming Zhang, Zhishan Zhang.

**Funding acquisition:** Tingjin Zheng, Chong Zeng, Zhishan Zhang.

**Investigation:** Tingjin Zheng, Yingzhi Xu, Zhishan Zhang.

**Methodology:** Jianming Zhang, Yingzhi Xu, Zhishan Zhang.

**Project administration:** Tingjin Zheng, Chong Zeng, Zhishan Zhang.

**Resources:** Yingzhi Xu, Chong Zeng.

**Software:** Tingjin Zheng, Chong Zeng.

**Validation:** Tingjin Zheng, Jianming Zhang, Yingzhi Xu, Chong Zeng, Zhishan Zhang.

**Visualization:** Yingzhi Xu, Chong Zeng, Zhishan Zhang.

**Writing – original draft:** Tingjin Zheng, Jianming Zhang.

**Writing – review & editing:** Tingjin Zheng, Rong Xu, Jianming Zhang, Yingzhi Xu, Chong Zeng, Zhishan Zhang.

## Supplementary Material




