# Supplementary material for: Prediction of renal cell carcinoma: Development and validation of machine learning model
Source: Medicine (Baltimore). 2026 Jan 23;105(4):e47205. doi: 10.1097/MD.0000000000047205 (PMC12851772; doi:10.1097/MD.0000000000047205)

Supplementary Fig. S1. Overall Feature Missing Status. (A). the completeness status of features across samples; (B)**.** the completeness rates of all enrolled features in HC and RCC.

A

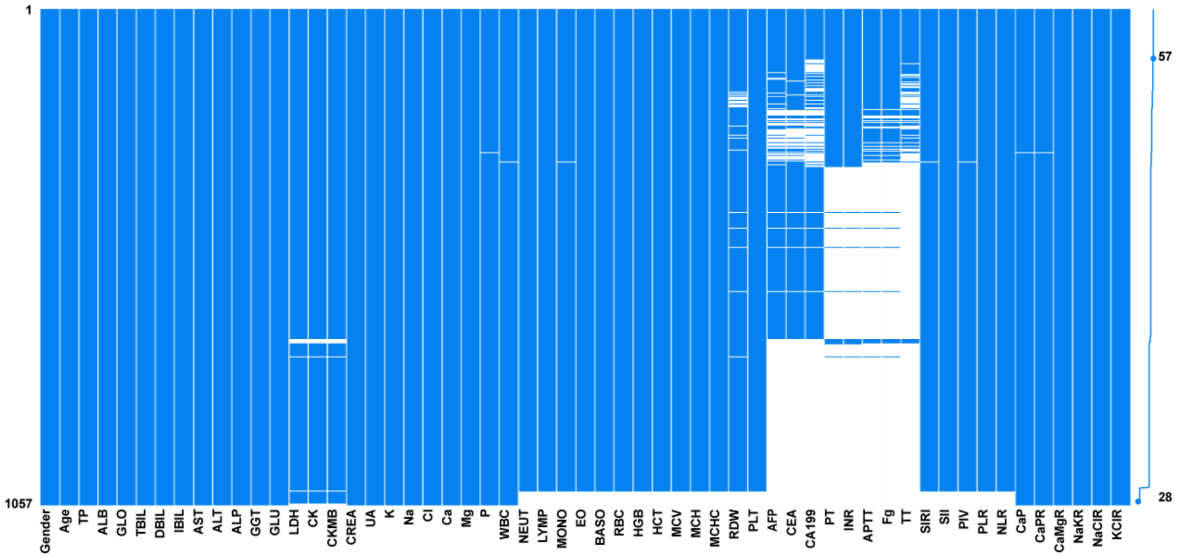

B

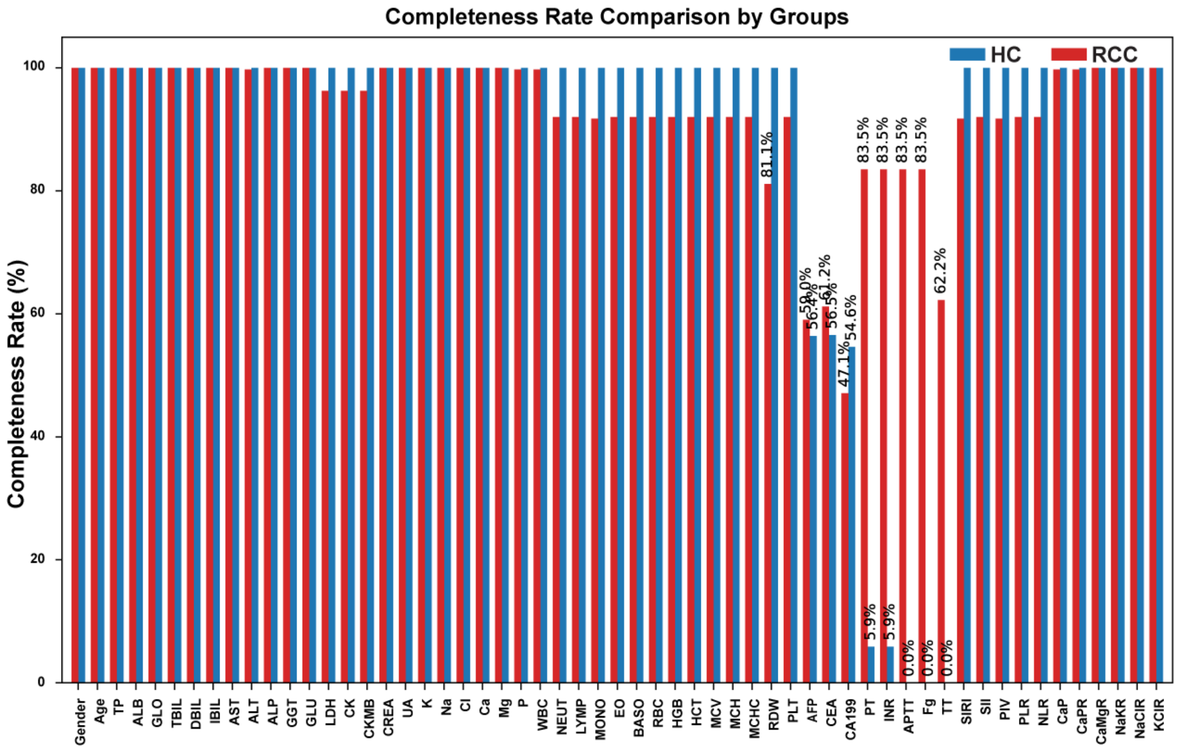

Supplementary Fig. S2. Correlation among univariate analysis selected features. (A). Heatmap demonstrates Correlation among univariate analysis selected 38 features. **(B).** hierarchical clustering analysis for univariate analysis selected 38 features.

A

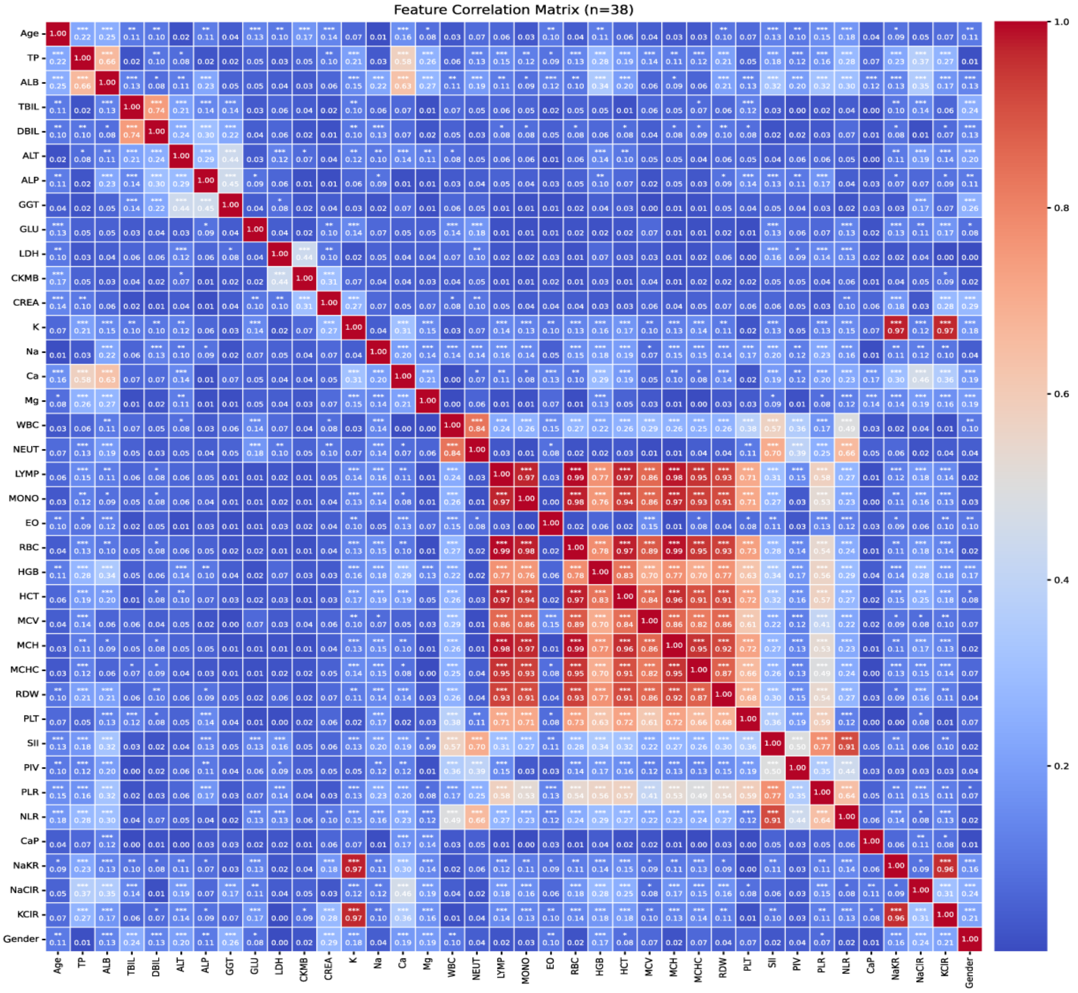

B

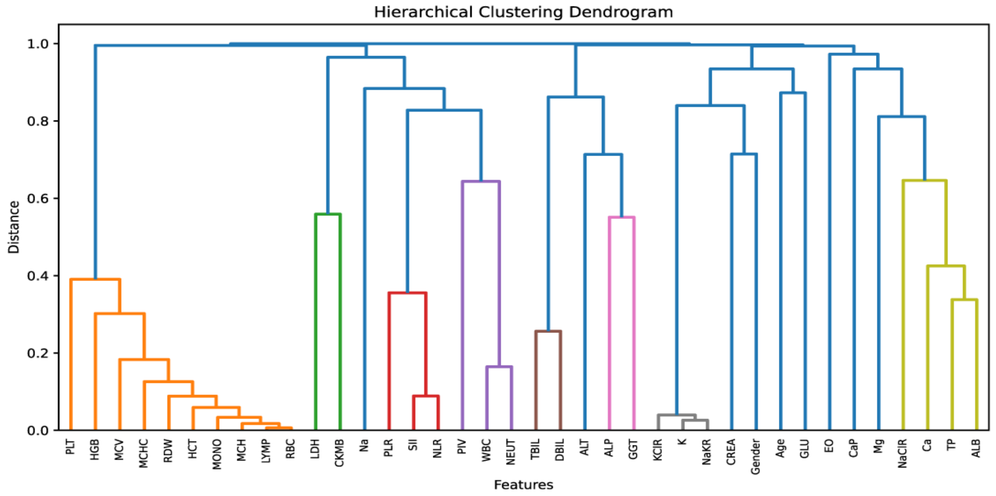

Supplementary Fig. S3. XGBoost-RFE for optimal features selection.

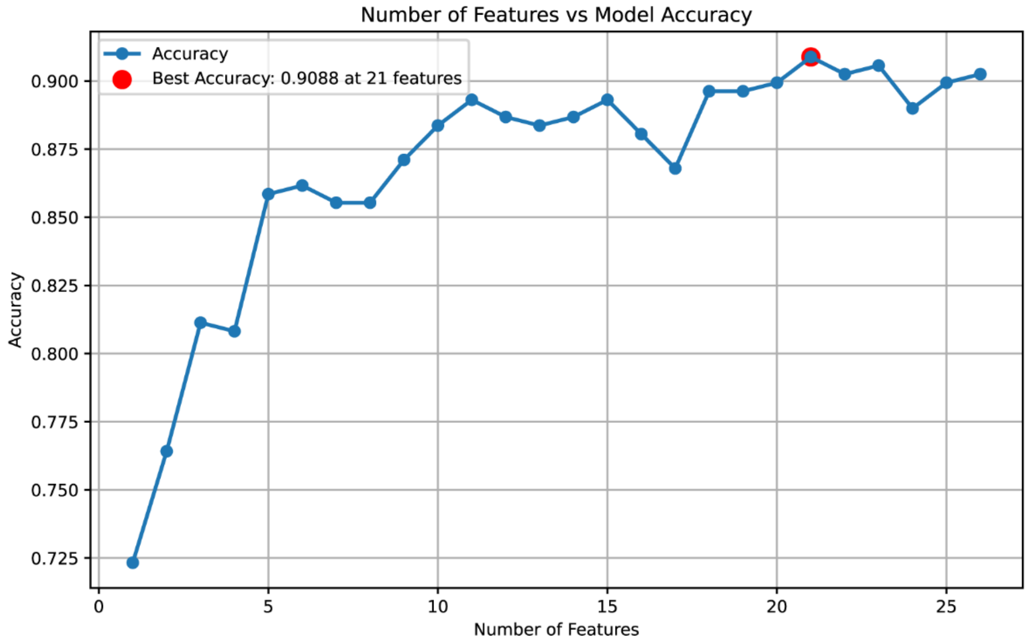

Supplement: Supplementary file 2 [file medi-105-e47205-s002.pdf]
